# Impact of COVID-19 pandemic and national lockdown in an orthopaedic and traumatology department—a comparison with the homologous period of 2019

**DOI:** 10.1097/j.pbj.0000000000000109

**Published:** 2021-01-18

**Authors:** Ana Ribau, João Vale, Francisco Xará-Leite, Ricardo Rodrigues-Pinto

**Affiliations:** a Department of Orthopaedics, Centro Hospitalar Universitário do Porto; b ICBAS-Instituto de Ciências Biomédicas Abel Salazar, Porto, Portugal.

**Keywords:** COVID-19, lock-down, orthopaedic surgery, pandemic

## Abstract

**Background:**

The coronavirus disease-2019 pandemic has forced health systems to undergo dynamic changes. This study aims to evaluate the impact of the pre-lockdown and of the lockdown period on the surgical activity of a Portuguese Orthopaedic and Traumatology Department and to compare it with the homologous period of 2019.

**Methods:**

The surgical activity between March 2 and May 2, 2020 and that of the homologous period of 2019 were analyzed and compared. Additionally, the impact of national and institutional measures was analyzed.

**Results:**

There was a decrease in elective surgeries, from 587 to 100. In 2020, 59.3% of all surgeries were urgent and 48.4% were trauma whereas in 2019 there were 25.5% urgent and 23.0% trauma surgeries (*P* < .001 and *P* < .001, respectively). There was no difference in the mean of proximal hip fractures operated per week (*P* = .310), even when analyzing only the lockdown period (*P* = .102). However, proximal hip fractures corresponded to significantly higher proportion of surgeries in 2020 (*P* = .04). Hand and tendon injuries significantly reduced in 2020, as were sports-related trauma surgeries. Mean number of days until surgery was significantly lower in 2020 (2020:1.6 ± 2.1, 2019: 2.2 ± 2.5, *P* = .012).

**Conclusion:**

Governmental and institutional measures had high impact on the production and on the epidemiology of trauma. While resumption of elective surgery is needed, lessons from these measures may help in the response to a possible second wave.

## Introduction

In December 31, 2019 Wuhan, China, reported a cluster of cases of pneumonia, identified as coronavirus.

The first case in Europe was reported on January 24, 2020 and in January 30, 2020, coronavirus disease-2019 (COVID-19) was declared a Public Health Emergency of International Concern by the World Health Organization.^1^ The COVID-19 epidemic rapidly spread out to the entire world and it was declared a pandemic on March 11. By this time there were 118,319 confirmed and 4292 deaths confirmed cases and death do to this cause around the world.^2^

The first case of COVID-19 in Portugal was confirmed on March 2 in Porto.^3^ Due to the devastating course of the disease worldwide, the Government of Portugal declared the State of Emergency on March 18, which was prolonged until May 2.^4^ Despite this effort, the virus widespread, and Portugal entered the Mitigation Stage on March 26.^5^

During this period, all public health care was forced to undergo dynamic changes. Centro Hospitalar Universitário do Porto, which is a public University Teaching Hospital and one of the 2 main Hospitals in Porto, the second largest Portuguese city, adjusted its clinical practice to allow for an adequate response to the high influx of patients suffering from COVID-19.

The Hospital Board instructed closure of the ambulatory center on March 14 and all elective surgeries were canceled from this day onwards until May 2. While some elective surgeries were performed between March 2 until March 14, from the later date onwards only urgent/emergent surgeries were performed. These included trauma (orthopaedic and spinal), infections, malignant tumors and non-traumatic spinal surgery with neurological impairment.

The Emergency State was revoked on May 2 and the country entered a phase of calamity situation. After this, the public health care tried to resume elective surgeries with the precautions imposed by the new reality.

The purpose of this paper was to evaluate the impact of the reorganization on the surgical activity in an orthopaedic surgery department during COVID-19 pandemic and to compare it with the homologous period of 2019.

Pandemic diseases, such as COVID-19, have a massive impact in health care systems. Information from this study is important not only to report but also it may help to understand the impact such a pandemic had on an orthopaedic department and help deal with future situations.

## Methods

Retrospective cohorts of cases from the hospital directory was performed. The surgical activity of the Orthopaedic and Traumatology Department of Centro Hospitalar Universitário do Porto between March 2 and May 2, 2020 was analyzed.

The orthopaedic and traumatology department consists of 62 beds, 6 of which are an intermediate care unit dedicated to spinal injury. On March 26, 37 beds were allocated to treat medical patients, with the remaining 25 beds being left for orthopaedic patients.

**Figure 1:**
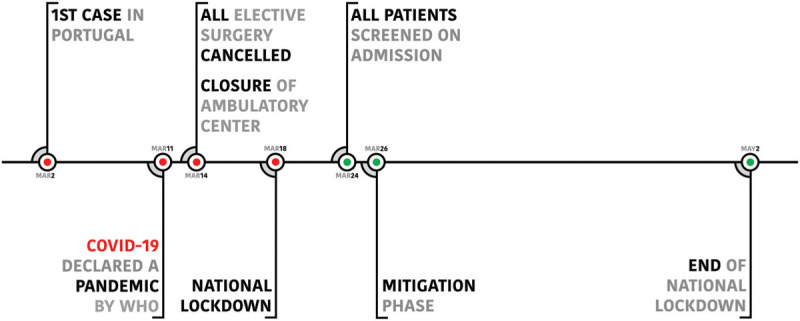
Timeline with government and hospital board implemented measures (WHO, World Health Organization).

Between March 2 and March 24, nasopharyngeal swabs were only performed in patients with clinical symptoms suspected of COVID-19. After that date, all patients admitted for surgery were screened for severe acute respiratory coronavirus 2 (SARS-CoV-2) with a nasopharyngeal swab for reverse transcription polymerase chain reaction test and waited for its result before ward or operating room admission. Figure 1 summarizes all the government and hospital board implemented measures

All surgeries performed were collected and divided into elective and urgent/emergent cases. The later consisted of trauma surgeries (fractures, dislocations, tendon and nerve injuries), bone and soft tissue malignant tumors and skeletal metastases and infections.

Demographic data was collected from all patients and included age, gender, American Society of Anesthesiology (ASA) classification, diagnosis, treatment, time until surgery and complications. Collected data was compared with the activity of the same department in the homologous period of 2019.

All SARS-Cov-2 positive patients were operated on a dedicated COVID-19 operating theatre, as previously described,^6^ which was available from March 24. The first surgery performed to a SARS-Cov-2 positive patient in the hospital was an orthopaedic procedure on March 31.

Whenever a negative patient developed symptoms suspected of COVID-19 another test was performed. All COVID-19 patients were admitted/transferred to a COVID-19 specific ward where they were followed by a team composed of an Internal Medicine/Infectious disease physician and the attending orthopaedic surgeon. COVID-19 patients were considered cured after 2 negative swabs. In case they were clinically stable to be sent home and had condition to be in isolation, they were discharged and the tests were performed as outpatient procedures.

The study was approved by the local ethics committee (REF: 2020.081 (064/DEFI/065-CE)) and is in accordance with the Declaration of Helsinki 2014.^7^

IBM^®^ SPSS^®^ Statistics was used for all the statistical analysis. The groups were compared using the Student's *t* test 2-tail (quantitative variables) or with the Fisher or Pearson chi-squared test (qualitative variables). Statistical significance was set at *P* < .05.

## Results

**Figure 2:**
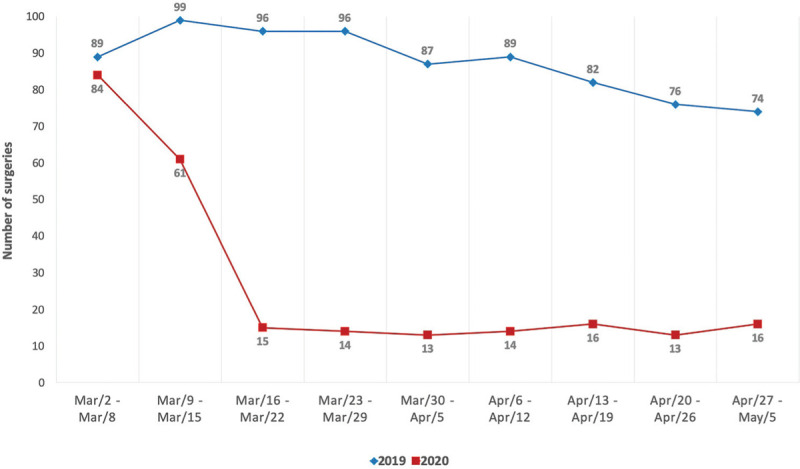
Comparison of the total of surgeries per week in 2019 and 2020.

Between March 2 and May 2, 2020, 246 orthopaedic and trauma surgeries were performed. In the homologous period of 2019, 788 surgeries were performed (Fig. 2).

**Figure 3:**
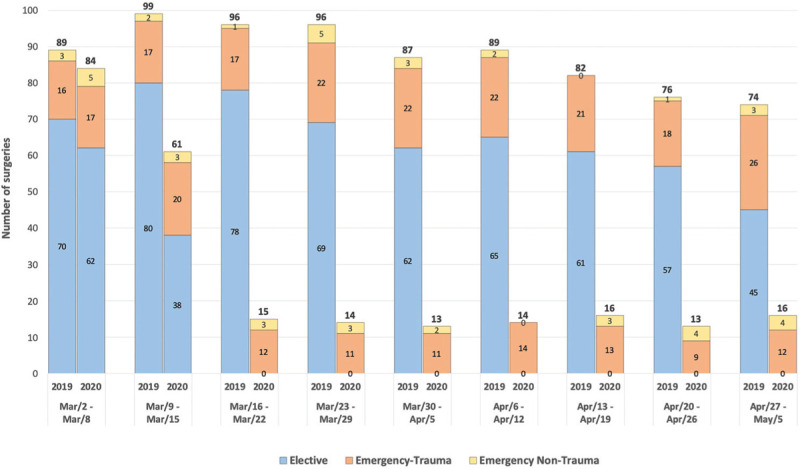
Comparison of surgical activity per week between 2019 and 2020.

In 2020, 100 of all surgeries performed (40.7%) were elective while in 2019 the number of elective surgeries was 587, representing 74.5% of all surgeries (*P* < .001). Additionally, 146 urgent and 119 trauma surgeries were performed in 2020, in contrast with 201 urgent and 181 trauma surgeries in 2019. Nonetheless, the preponderance of these surgeries was significantly higher in 2020, with 59.3% urgent and 48.4% trauma surgeries in 2020 versus 25.5% urgent and 23.0% trauma surgeries in 2019 (*P* < .001 and *P* < .001, respectively). When analyzing the lockdown period, in which no elective surgeries were performed and when people were instructed to stay at home, a mean of 14.4 ± 1.3 trauma surgeries per week were performed, a significant decrease from the 22.3 ± 4.0 trauma surgeries per week in 2019 (*P* < .001) (Fig. 3).

All elective surgeries performed in 2020 occurred between May 2 and 14, after which only urgent/emergent cases were operated. The details of the elective surgeries performed in 2019 and 2020 are depicted in Table 1.

**Table 1:** Elective surgeries in 2019 and 2020

Type of surgery	2020	2019
Spine	10	49
Hand and elbow	28	178
Shoulder	6	38
Hip	12	69
Knee	17	119
Foot and ankle	14	69
Benign soft tissue and bone tumors	7	31
Chronic infection	1	13
Removal of osteosynthesis material	5	21

There were 146 urgent surgeries in 2020, a slight decrease from the 201 performed in the same period of 2019. The urgent surgeries in 2020 comprised 119 trauma (fractures, dislocations and tendon and nerve injuries) (Table 2), 12 acute infections (10 post-operative osteosynthesis or arthroplasty infections, 1 knee and 1 ankle septic arthritis) 8 malignant tumors, 3 chronic infections (1 spinal and 2 peri-prosthetic), 1 biopsy for an odontoid tumour, 1 lumbar disc herniation with acute neurological impairment, 1 cerebrospinal fluid fistula, and 1 second-stage phalanx lengthening procedure in a patient with acrosyndactyly.

**Table 2:** Trauma surgeries by diagnosis

	2020 Pre-lockdown (n = 37)	2020 Post-lockdown (n = 82)	2019 (n = 181)	2020 Pos-lockdown vs 2019, *P*
Spine				
Unstable spinal fracture	2 (5.4%)	7 (8.5%)	10 (5.5%)	.114
Upper limb	6	12	53	
Glenoid fractures	(0%)	0 (0%)	1 (0.6%)	1.000
Proximal humerus fractures	3 (8.1%)	4 (4.9%)	13 (7.2%)	.481
Humeral shaft fractures	0 (%)	0 (0%)	3 (1.7%)	.554
Elbow fractures	1 (2.7%)	2 (2.4%)	3 (1.7%)	.649
Forearm fractures	0 (%)	1 (1.2%)	3 (1.7%)	.784
Distal radius fractures	0 (%)	3 (3.7%)	11 (6.1%)	.403
Hand injuries^∗^	2 (5.4%)	2 (2.4%)	19 (10.5%)	**.026** (OR 0.213, CI 95% 0.048–0.938)
Lower limb	32	62	118	
Pelvic ring fractures	0 (%)	2 (2.4%)	1 (0.6%)	1.000
Periprosthetic fractures	0 (%)	2 (2.4%)	2 (1.1%)	.591
Proximal hip fracture	20 (54.1%)	41 (50%)	71 (39.2%)	.102
Femoral shaft fractures	1 (2.7%)	2 (2.4%)	2 (1.1%)	.591
Distal femur fracture	2 (5.4%)	1 (1.2%)	1 (0.6%)	.527
Patella fractures	0 (0%)	0 (0%)	1 (0.6%	1.000
Tibial plateau fractures	2 (5.4%)	2 (2.4%)	2 (1.1%)	.591
Tibial shaft fractures	0 (%)	2 (2.4%)	11 (6.1%)	.207
Ankle fractures	5 (13.5%)	10 (12.2%)	18 (9.9%)	.667
Achilles tendon ruptures	1 (2.7%)	0 (0%)	7 (3.9%)	**.053**
Fractures of the foot	0 (0%)	1 (1.2%)	2 (1.1%)	.230

∗ Hand injuries with or without fractures and with or without tendinous injuries.

There were differences in the type of trauma surgeries performed in 2020 comparing to 2019 (Table 2).

The mean age of patients undergoing trauma surgery did not differ between years (2020: 70.7 ± 19.8 years and 2019: 67.1 ± 20.5 years, *P* = .179). There were 79 (66.4%) females in 2020 and 117 (58.2%) in 2019. The ASA score distribution was similar between the 2 periods (2020: 83% ASA II or ASA III and 2019: 80.4% in 2019, *P* = .462).

During the analyzed period in 2020, a mean of 6.6 ± 2.5 proximal hip fractures were operated per week, no significant decrease from the 7.8 ± 2.5 operated in 2019 (*P* = .310). Even concerning only the lockdown period, there was no significant decrease in the mean number of proximal hip fractures performed per week (2020: 5.9 ± 1.7 and 2019: 7.8 ± 2.5, *P* = .102). Although the slight decrease in absolute number, preponderance of proximal hip fractures significantly increased in 2020 (2020: 61/119 (51.3%) and 2019: 71/181 (39.2%), *P* = .04).

During the lockdown period, 2 (2.4%) hand traumas were operated, a significant decrease from the 19 (10.5%) performed in 2019 (*P* = .026, OR 0.213, CI 95% 0.048–0.938). In this period, no Achilles tendon ruptures were operated, compared to 7 (3.9%) in 2019 (*P* = .053)

The mean number of days until surgery was 1.6 ± 2.1 days in 2020 representing a significant decrease from the 2.2 ± 2.5 days in 2019 (*P* = .012).

During the analyzed period, 78 nasopharyngeal swabs were performed prior to surgery—all patients that were operated after March 26 plus urgent surgeries that were not admitted through the emergency department before that day. One hundred and sixty-eight patients had not been screened prior to admission but 4 developed respiratory symptoms during hospital stay or contacted with a patient known to be COVID-19 positive. Of these, 3 were confirmed to be SARS-CoV-2 positive. Of those 3 patients, 2 had been submitted to femoral nailing due to intertrochanteric fractures and were sharing a ward, and 1 had been submitted to open reduction and internal fixation of a malleolar fracture. After COVID-19 diagnosis they were immediately transferred to a COVID-19 ward. From the patients screened before admission (78), 2 were SARS-CoV-2 positive. These corresponded to an intertrochanteric fracture and a tibial plateau fracture. During this period, no patients had a clinical condition that required surgery without the test result.

All 5 patients confirmed to be SARS-CoV-2 positive (2 before and 3 after surgery) had uneventful post-ops. Table 3 depicts the details of those patients.

**Table 3:** COVID-19 trauma patients

Gender/age	Diagnosis	Surgery	Time to surgery (d)	COVID-19 symptoms	Complications
Female/83	Bimalleolar fracture	Open reduction, internal fixation	0	None	None
Female/89	Intertrochanteric fracture	Cephalomedullary nail	0	Fever	None
Female/98	Intertrochanteric fracture	Sliding hip compression screw	0	None	None
Female/87	Intertrochanteric fracture	Cephalomedullary nail	1	None	None
Male/57	Tibial plateau fracture	Open reduction, internal fixation	1	None	None

## Discussion

This is a retrospective study and therefore possibly has the biases associated with the analysis is performed post-hoc. The information bias is a major limitation although we tried to control the selection bias by comparing demographic data between groups.

In the present study, the COVID-19 Pandemic is shown to have had a significant impact on the surgical activity of and orthopaedic and traumatology department. There was almost a 6-fold decrease in the number of elective surgeries, from 587 to 100. This fact was deemed necessary due to the extreme situation the health services had to face, in order to provide more resources to treat COVID-19 patients, but also to avoid the spread of the virus.^8^

While some elective surgeries were performed after the 1^st^ Portuguese reported case, in March 2, 2020, all elective surgeries were canceled after March 14, 2020. From that period onwards only urgent and emergent surgeries were performed with all other orthopaedic procedures being postponed. The surgeries that were not postponed in our department are according to the recommendations for triaging patients, as described by Randelli and Compagnoni^9^ and Cardoso and Rodrigues-Pinto.^10^

As there were shortage of ICU beds and blood units for transfusion, non-trauma cases requiring surgery had to be triaged and all surgeries in which ICU admission or transfusion need were predicted were postponed, if possible. Three chronic infections were considered prioritary/urgent and were operated during this period. In neither of these ICU admission nor extensive blood loss were predicted and required.

The total number of urgent surgeries decreased from 201 in 2019 to 146 in 2020. Regarding only trauma surgeries there was a decrease from 181 in 2019 to 119 in 2020. This is possibly due to a reduction in all types of activity (work or sports), as the major decrease was verified in hand-trauma and in Achilles tendon ruptures, whereas proximal hip fractures still had similar frequency and increased in preponderance. Hampton et al also show that during the lockdown in United Kingdom the orthopaedic trauma decreased with no change in fragility fractures.^11^ There was a significant reduction in the number of hand injuries, which mostly result from work trauma and in the number of sports-related injuries, such as Achilles tendon ruptures during the COVID-19 pandemic with no change on the impact of fragility fractures.

The time to surgery decreased in 2020, probably because there was no elective activity, and so, all the efforts were focused on the quick resolution of urgent patients, in order to avoid prolonged hospitalizations and reduce the risk of acquiring SARS-CoV-2 infection.

Two main messages can be drawn from this study. First, to combat the COVID-19 pandemic, the Portuguese Government and the Hospital Board took serious measures (particularly the institution of lockdown and the cancelation of all elective surgeries) to reduce contamination and to prepare hospitals to treat COVID-19 patients. This may be one of the reasons Portugal has 125.37 deaths per million on May 23, 2020, a much lower number than most European countries, the United States of America and Canada.^12^ Unfortunately, elective patients who were postponed still have to be treated, and the consequences of this delay may be harmful to them and overload a system that recently suffered from an economic crisis with a cut in health expenditures.^13^ Thus, in May 2, 2020 the government recommended to resume elective surgery according to the available resources, providing that all patients admitted for elective surgery are previously screened for SARS-CoV-2, and only those with negative results are admitted for surgery, which is in line with the most recent guidelines.^14^ The second message from this study relates to the type of trauma. While the admissions to the Orthopaedic and Traumatology Emergency Department have drastically reduced (data presented in a different publication), the number of trauma requiring surgery has only slightly decreased, with the most striking difference being seen in the type of trauma.

Data presented in this study highlights the dynamic changes over a period of 2 months to provide appropriate response to the COVID-19 pandemic, while still providing orthopaedic and traumatology care to urgent and emergent patients. However, since it is monocentric, relatively small numbers of patients were included and some changes in the epidemiology may have been missed due to their low frequency. A multicenter study would be more effective at identifying all changes that happened in Portugal during the lockdown.

Information from this study may help to understand the impact such a pandemic had on an orthopaedic department. While some authors suggest there may be a second wave of infection,^15^ this knowledge may prove fundamental in its timely containment and resolution.

## Conclusion

The COVID-19 pandemic has had a high impact in this orthopaedic surgery department. The governmental measures probably affected the demography of the trauma patients, with a reduction of work and sports-related trauma but a maintenance of fragility fractures. The reorganization allowed a quicker response to the trauma patients. Nonetheless, the delay of elective surgeries raises concerns as, many orthopaedic patients remain untreated, putting pressure on a public health care system which was already overloaded before this pandemic. Thus, resumption of elective surgery is needed since it is essential to assure a well-functioning health care.

## Conflicts of interest

The authors have declared no conflict of interest.
